# Overexpression of *DYRK1A*, a Down Syndrome Candidate gene, Impairs Primordial Germ Cells Maintenance and Migration in zebrafish

**DOI:** 10.1038/s41598-017-15730-w

**Published:** 2017-11-10

**Authors:** Yanyan Liu, Ziyuan Lin, Mingfeng Liu, He Wang, Huaqin Sun

**Affiliations:** 1 Prenatal Diagnosis Center, Department of Obstetrics & Gynecologic, Key Laboratory of Birth Defects and Related Diseases of Women and Children (Sichuan University), Ministry of Education, West China Second University Hospital, Sichuan University, Chengdu, 610041 People’s Republic of China; 2 SCU-CUHK Joint Laboratory for Reproductive Medicine, Key Laboratory of Birth Defects and Related Diseases of Women and Children (Sichuan University), Ministry of Education, Department of Pediatrics, West China Second University Hospital, Sichuan University, Chengdu, 610041 People’s Republic of China; 3 Key Laboratory of Bio-resource and Eco-environment of Ministry of Education, College of Life Science, Sichuan University, Chengdu, Sichuan 610064 People’s Republic of China

## Abstract

*DYRK1A*, located on chromosome 21, is a major candidate gene of Down syndrome (DS, trisomy21), and its overexpression is associated with abnormal phenotype of Down syndrome patients. The defects of gonads and germ cells in Down Syndrome suggest that overexpression of *DYRK1A* has potential effect on primordial germ cells (PGCs) development. Human and zebrafish *DYRK1A* protein sequence possess 75.6% similarity and same function domains, suggesting the evolutional conservation. Here, we used zebrafish model to detect the definite role of excessive expression of *DYRK1A* in PGCs development during embryogenesis. We injected *DYRK1A* mRNA into embryos and detected the PGCs marker gene *vasa* and *nanos1*. Results showed depletion in numbers and disordering migration of PGCs in human or zebrafish *DYRK1A* overexpressed zebrafish embryos. Quantitative proteome analysis indicated that embryonic proteins were significantly altered in *DYRK1A* overexpressed embryos. Of note, ca15b and piwil1, two identified critical factors for PGCs development, showed ectopic expression induced by overexpressed *DYRK1A*. In brief, we demonstrate that overexpression of *DYRK1A*, a candidate gene of Down’s syndrome, impairs PGCs development during early embryogenesis by altering key factors in embryos. Importantly, our work may provide a conceivable mechanism for the gonads and germ cells defects of Down syndrome patients.

## Introduction

In 1966 of last century, Finch *et al*. reported that the chromosomal aberration leads to sub- or infertility in carriers of trisomy 21, that is, Down syndrome^1^. Hojager *et al*.^2^ showed a reduced follicle number and a retarded follicle growth in prepubertal female patients gonads^2^. The tubuli seminiferi contain a reduced number of germ cells or are completely devoid of germ cells in the testes of adult individuals^3,4^, which caused by aneuploid conditions meiotic defects^5,6^. Leffler *et al*. in year 1999 showed migration delay and reduction of PGCs in trisomy 16 mouse^7^, an animal model for Down’s syndrome.

*DYRK1A* gene in human maps to the Down syndrome critical region q22.2 of chromosome 21^8–12^. *DYRK1A* plays pivotal regulatory roles in the signaling of cell proliferation and development, which has dual substrate specificities. Autophosphorylation for self-activation takes place on the tyrosine-321 residue in the active loop of the catalytic domain^13,14^, and target protein phosphorylation occurs on serine/threonine residues^15^. *DYRK1A* has been reported to phosphorylate or interact with several proteins, including STAT3, FHKR, Gli-1, eIF2Be, Tau, dynamin, glycogen synthase, 14-3-3, CREB, cyclin L2, Arip4, Hip-1, PAHX-AP1, and SF3b1, suggesting that *DYRK1A* participates in multiple biological pathways by diverse array of interactions^15–17^.

Compared with the healthy person, the expression of *DYRK1A* in Down Syndrome patients shows increased pattern^18^. The many features of Down’s syndrome include neurological, skeletal, cardiovascular and immunological defects, and are generally thought to originate from a 1.5-fold increase in the dosage of genes, including *DYRK1A*, within a critical region of chromosome 21, which is present in triplicate in all cases of Down’s syndrome^19^. The defects of gonads and germ cells in Down Syndrome patients suggest that overexpression of *DYRK1A* has potential function on primordial germ cells (PGCs) development. Importantly, the definite role of increased dosage of *DYRK1A* in PGCs development remains indistinct.

Model organism zebrafish is relevant to higher vertebrates significantly, with highly manageable genetic manipulation, which making it an exceptional animal model for exploring molecular mechanism regulating key developmental processes. At very early embryogenesis, zebrafish PGCs are segregated from the somatic lineage, and start a characteristic performance of migration toward the genital ridges shortly thereafter^20,21^. This migration process is completed within the first developmental day, and can be detected at high resolution using simple microscopy, due to the optical transparency of zebrafish embryos. Hence, zebrafish embryo has been considered to be excellent *in vivo* model for investigation of PGC migration^22^.

Zebrafish PGCs are specified in different locations in the embryo and migrate toward the location where the gonad develops, the site where they eventually differentiate into gametes, sperm and egg. Following their specification during early embryonic stages, PGCs polarize and acquire motility. As they migrate, PGCs are presented with attractive and repulsive guidance cues provided by somatic cells along the migration path^23^, which exist complicated developmental and cellular mechanisms. Some key factors play critical role for this migration process, such as Piwil1 and Ca15b^23–25^.

Here, we used zebrafish model and two PGCs markers *vasa* and *nanos1* to investigate the function of overexpression *DYRK1A* on PGCs during embryo development. Results showed that PGCs in *DYRK1A* overexpressed embryos were decreased and disordered. Alone with the PGCs defect, critical factors for PGCs development were altered in aberrant *DYRK1A* injected embryos, suggesting the important and definite role of *DYRK1A* for PGCs development in early embryogenesis.

## Results

### *DYRK1A* protein is evolutionally conserved between zebrafish and human

**Figure 1:**
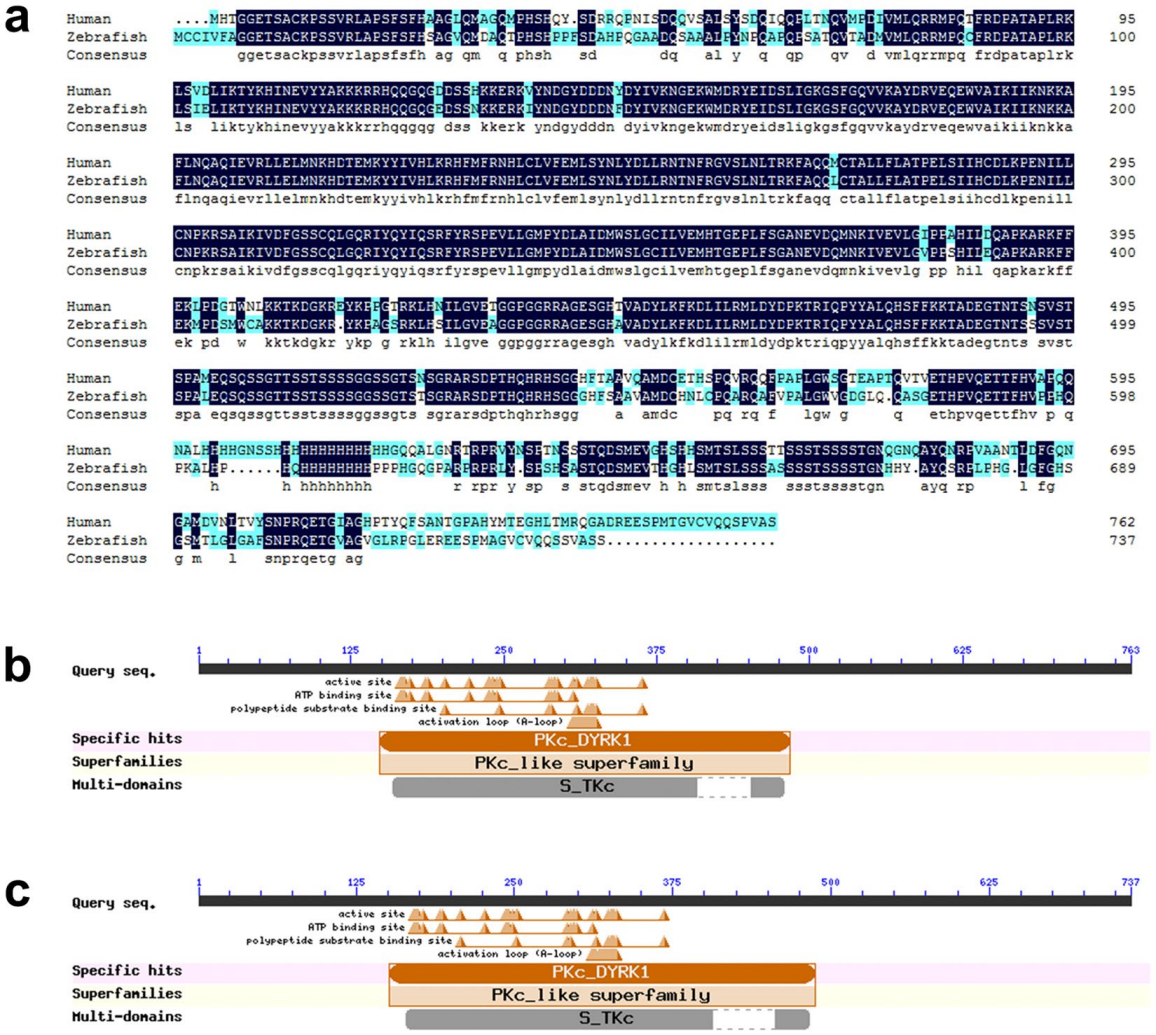
*DYRK1A* is evolutionally conserved between human and zebrafish. (**a**) Complete sequence alignment of human *DYRK1A* and zebrafish *DYRK1A* protein sequence. Conserved domain of human *DYRK1A* (**b**) and zebrafish *DYRK1A* (**c**) protein analyzed by NCBI conserved domains website.

Zebrafish *DYRK1A* protein, similar to human *DYRK1A*, shows 75.6% similarity to its human ortholog **(**Fig. 1a **)**. Particularly, they have same PKc_DYRK1 and S_TKc domains which are important for *DYRK1A* function **(**Fig. 1b and c **)**. The evolutional conservation suggests the critical role of *DYRK1A* for vertebrate and qualification of zebrafish as model organism to study *DYRK1A* molecular function.

### Expression pattern of *dyrk1a* during zebrafish early embryogenesis

**Figure 2:**
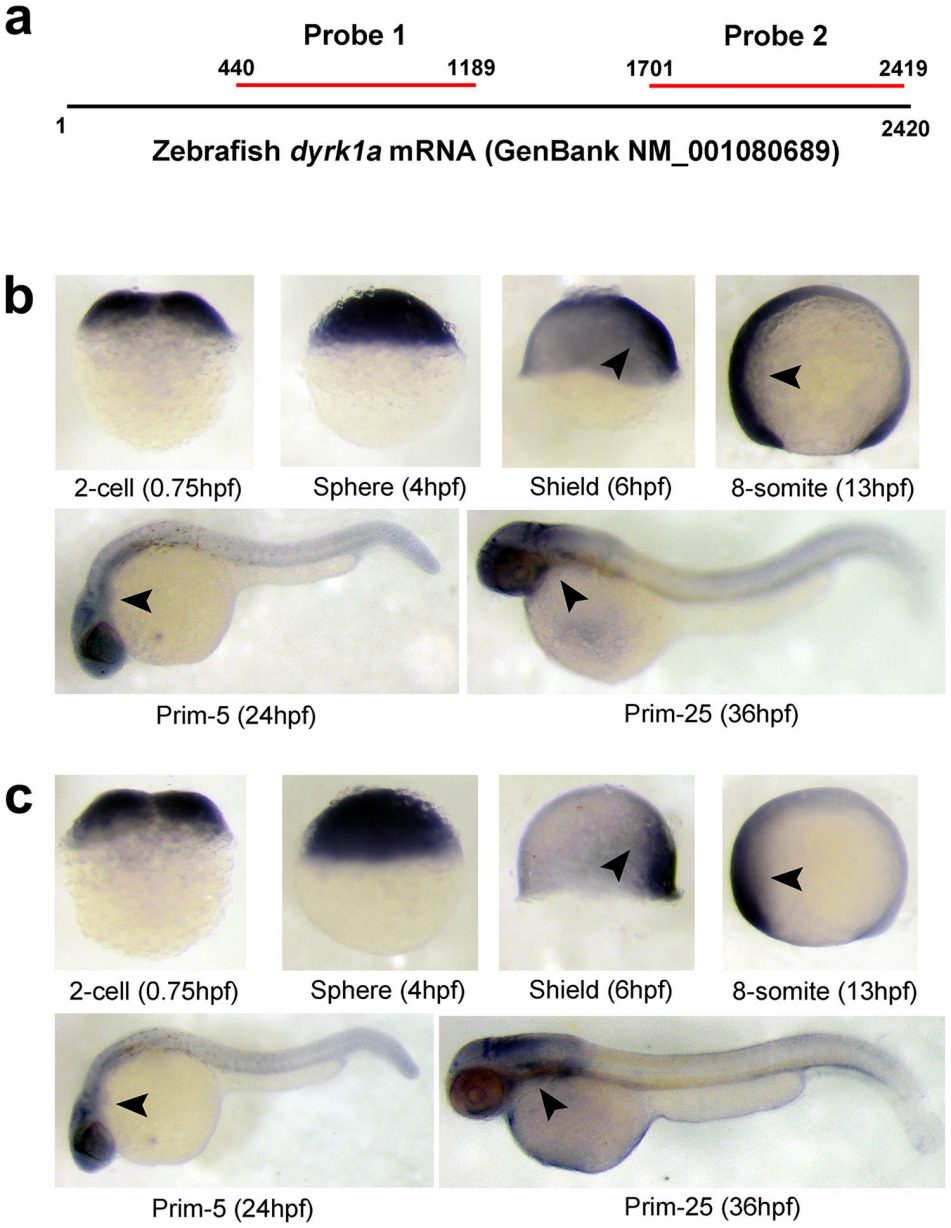
Spatiotemporal expression pattern of *DYRK1A* transcript in zebrafish embryos. (**a**) Location of anti-sense RNA probe for detection of zebrafish *DYRK1A* using WISH. (**b**) Detection by Probe 1 at indicated stages. (**c**) Detection by Probe 2 at indicated stages. Arrows show the stronger expression region of *DYRK1A*. The same expression pattern of *DYRK1A* shown by two non-overlapping probes, demonstrating the specificity and identical spatially restricted expression patterns of *DYRK1A* in zebrafish early embryogenesis. Embryo orientations: 2-cell and Sphere stage, lateral views with the animal pole oriented at the top; Shield stage, lateral view with the dorsal side oriented at the right; 8-somite, Prim-5 and Prim-25 stage, lateral views with anterior oriented toward the left.

Expression of *DYRK1A* mRNA during zebrafish embryogenesis was examined by whole mount *in situ* hybridization (WISH), and two nonoverlapping probes for *DYRK1A*, localizing at positions 440–1189 bp (probe 1) and 1701–2419 bp (probe 2) (Fig. 2a **)**, were used to demonstrate specificity and obtain identical spatially restricted expression patterns^26^.

Results show that zebrafish *DYRK1A* mRNA appears in the 2-cell stage and in all blastodermal cells until the dome stage (Fig. 2b and c). At the onset of gastrulation, *DYRK1A* expression starts to concentrate on the dorsal side **(**Fig. 2b and c, Shield stage). When segmentation starts, *DYRK1A* is expressed in axis ubiquitously and shows stronger expression at anterior part of embryo **(**Fig. 2b and c, 8-somite stage), and then branchial and pharyngeal arches gradually gain stronger expression **(**Fig. 2b and c, Prim-5 and Prim-25 stage).

Next, expression of *DYRK1A* protein during zebrafish embryogenesis was examined by using whole-mount immunohistochemistry (WIHC). Results of WIHC show that spatiotemporal expression pattern of *DYRK1A* protein is similar to *DYRK1A* mRNA (Supplemental Figure S1). These results of expression pattern suggest potential and important role of *DYRK1A* in early embryogenesis, especially at blastula period of beginning of PGCs development.

### Overexpression of *DYRK1A* impairs PGCs Maintenance and Migration

**Figure 3:**
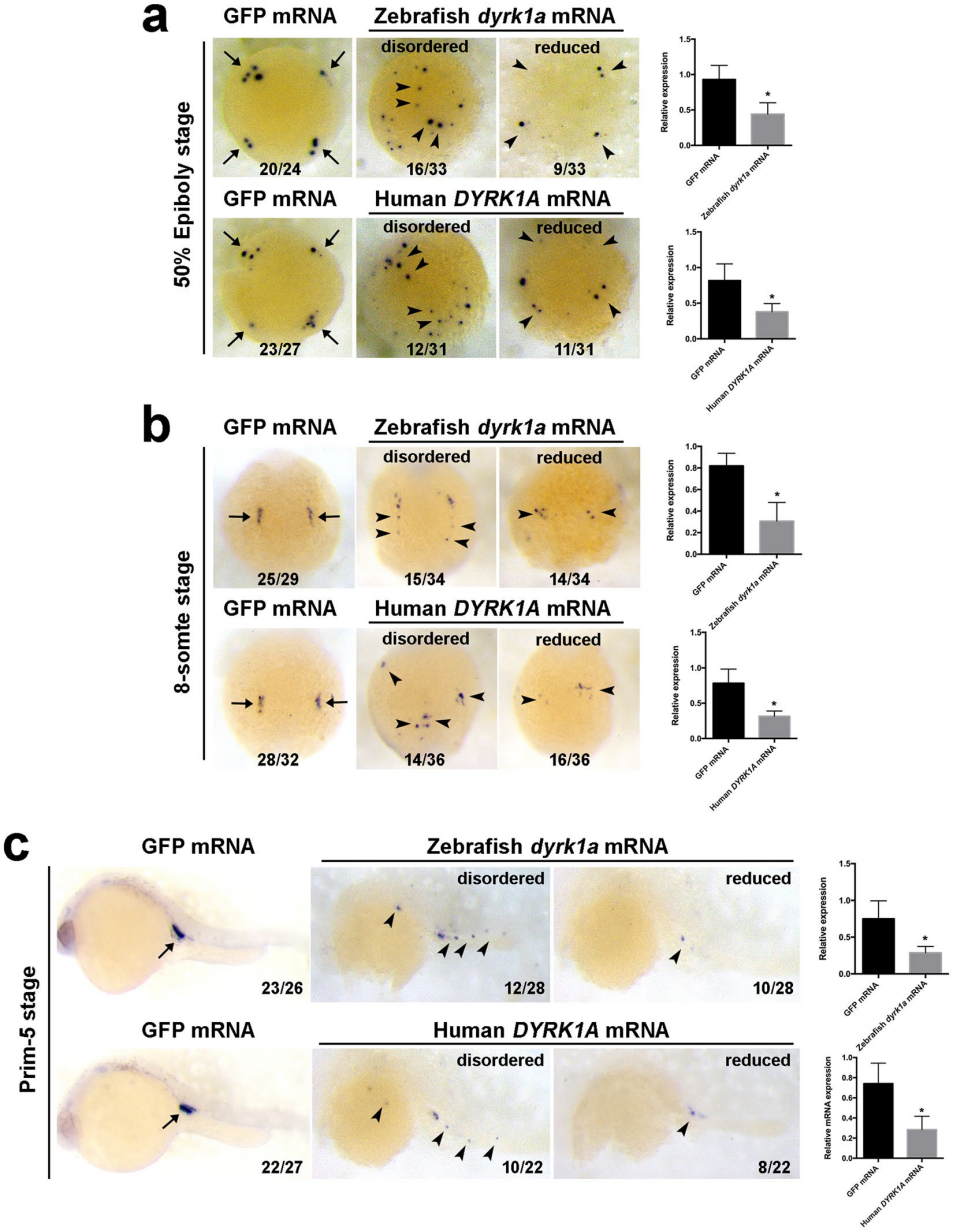
Overexpression of *DYRK1A* induces *nanos1*-marked PGCs deficiency in early zebrafish embryo. Analysis of localization and strength of *nanos1* positive cells in *DYRK1A* overexpressed embryos by WISH at 50% Epiboly stage (**a**), 8-somite stage (**b**) and Prim-5 stage (**c**). Histogram representing the relative expression detected by qPCR in GFP injected and *DYRK1A* overexpressed embryos at corresponding assay. Embryo orientations: 50% Epiboly stage, top view with the dorsal oriented at the right; 8-somite, dorsal view with anterior oriented at the top; Prim-5 stage, lateral views with anterior oriented toward the left. Arrows show the normal location of PGCs, arrowheads demonstrate the aberrant position of PGCs induced by overexpressed *DYRK1A*. The numbers indicated in each picture are the number (left) of affected embryos with phenotype similar to what is shown in the picture and the total number (right) of observed embryos. The same number labeling was used thereafter.

**Figure 4:**
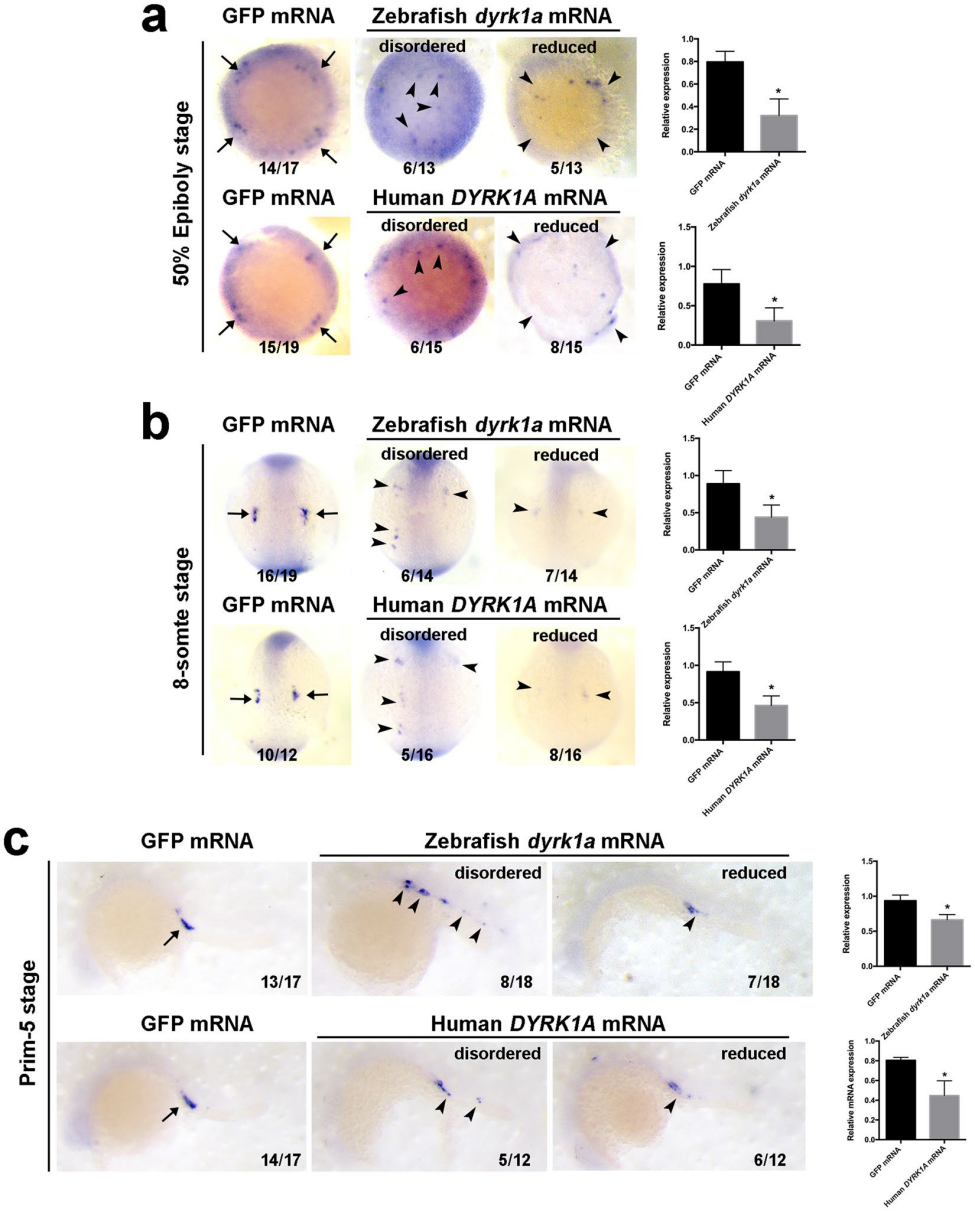
Overexpression of *DYRK1A* induces *vasa*-marked PGCs deficiency in early zebrafish embryo. Analysis of localization and strength of *vasa* positive cells in *DYRK1A* overexpressed embryos by WISH at 50% Epiboly stage (**a**), 8-somite stage (**b**) and Prim-5 stage (**c**). Histogram representing the relative expression detected by qPCR in GFP injected and *DYRK1A* overexpressed embryos at corresponding assay. Embryo orientations: 50% Epiboly stage, top view with the dorsal oriented at the right; 8-somite, dorsal view with anterior oriented at the top; Prim-5 stage, lateral views with anterior oriented toward the left. Arrows show the normal location of PGCs, arrowheads demonstrate the aberrant position of PGCs induced by overexpressed *DYRK1A*.

To determine whether *DYRK1A* overexpression effects on maintenance and migration of the PGCs, we followed the overexpression process in embryos in which the *DYRK1A* mRNA was injected. Since human and zebrafish *DYRK1A* protein sequence and domain are evolutionally conserved, we injected human and zebrafish *DYRK1A* mRNA individually to zebrafish embryos to investigate whether *DYRK1A* has conserved effect to PGCs development. PGCs were marked by gene *nanos1* and *vasa* at 50% epiboly, 8-somite and Prim-5 stage, and we found that overexpression of human and zebrafish *DYRK1A* individually led to same abnormity of PGCs, showing ectopic locations and reduction (Figs 3 and 4), suggesting that human and zebrafish *DYRK1A* functions conservatively to PGCs development. To confirm the importance of *DYRK1A* conserved domain PKc in PGCs development, we constructed deletion mutants of *DYRK1A* lacking PKc domain. Embryos injected with mRNA of *DYRK1A* PKc domain deletion mutant showed that the mutation abrogated the abnormality of PGCs development (Supplemental Figure S2), suggesting the requirement of PKc domain for *DYRK1A* function.

Furthermore, to investigate role of *dyrk1a* deficiency for PGCs development, we used *DYRK1A* popular inhibitor Epigallocatechin Gallate (EGCG) to treat WT embryos. Results showed that EGCG could not lead to PGCs abnormality, even though we tried wide range of concentration from 10 µm to 200 µm (Supplemental Figure S3). Taken together, similar to the situation of Down syndrome, *DYRK1A* with overexpressed state, but not deficiency, leads to abnormality of PGCs, depending on its PKc conserved domain.

### Proteomics analysis shows aberrant expression of key factors essential for PGCs development in *DYRK1A* overexpressed embryos

**Figure 5:**
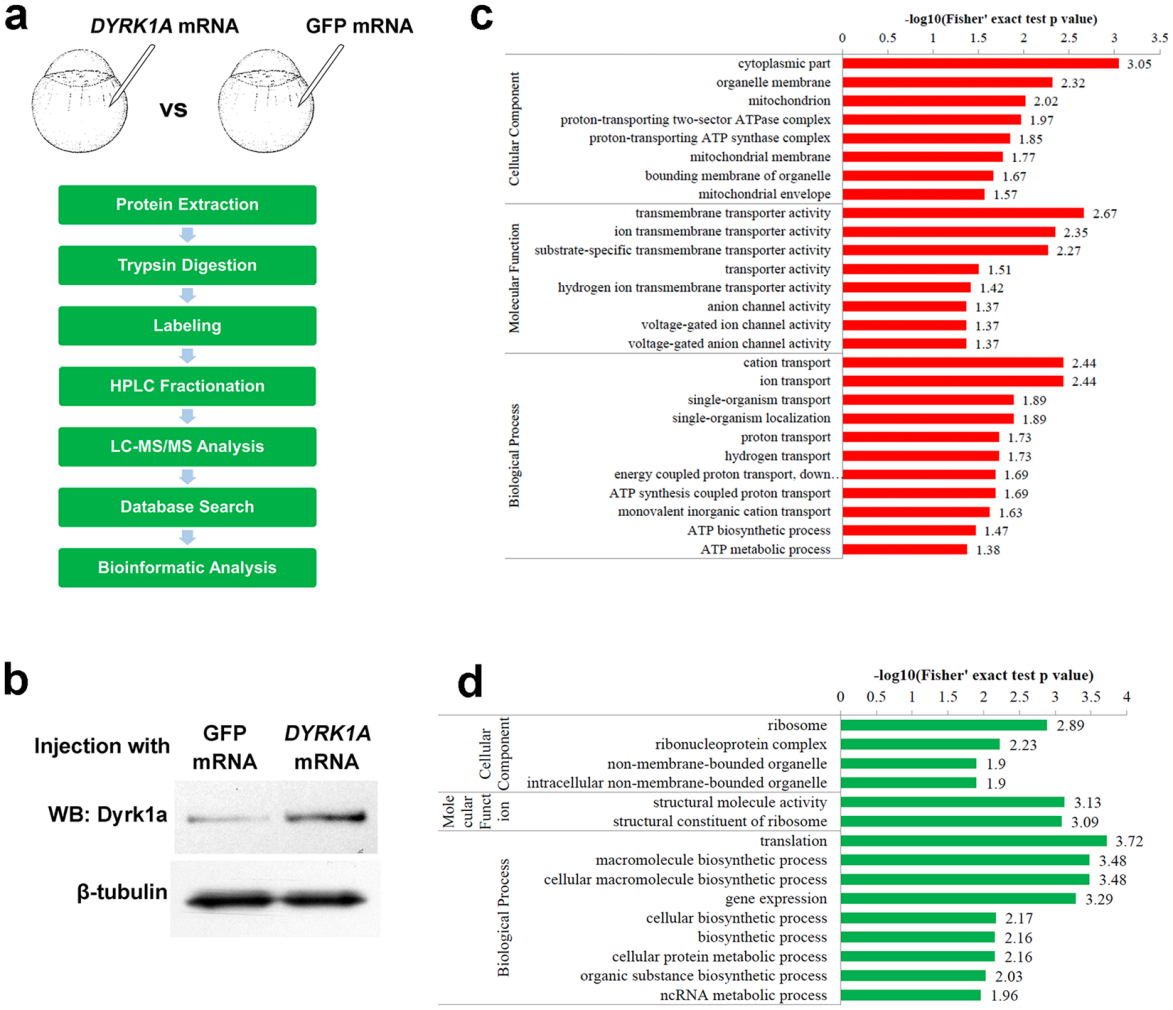
Proteomics analysis shows the altered proteins pattern essential for PGCs development in *DYRK1A* overexpressed embryos. (**a**) General work flow of Proteomics analysis in this work. (**b**) Embryos injected with *DYRK1A* mRNA show increased protein level of *DYRK1A*. The blots shown are cropped; the uncropped full-length gels are presented in the Supplementary Figure 1. Gene Ontology (GO)-based enrichment analysis of up-regulated (**c**) and down-regulated (**d**) proteins (*DYRK1A* mRNA vs GFP mRNA).

To discover the molecular mechanism of *DYRK1A* overexpression regulating PGCs development, we used an integrated approach involving TMT labeling and LC-MS/MS to quantify the dynamic changes of the whole proteome of zebrafish embryos at 50% epiboly stage (injection of human *DYRK1A* mRNA vs GFP mRNA) **(**Fig. 5a **)**. Western blot assay shows the increased protein level of *DYRK1A* in the embryos injected with *DYRK1A* mRNA **(**Fig. 5b and Supplemental Figure S4). In total, 1,573 proteins from embryos were identified in response to *DYRK1A* mRNA and GFP mRNA injection, among which 1,324 proteins were quantified. All the annotation and quantification information were presented in the Supplemental Table S1. Relative quantitation of proteins was divided into two categories. Quantitative ratio over 1.2 was considered up-regulation while quantitative ratio less than 1/1.2 was considered as down-regulation. Results showed that *DYRK1A* mRNA injection induced 265 differentially expressed proteins (141 up-regulated and 124 down-regulated).

To characterize the function of these altered proteins, Gene Ontology (GO)-based classification analysis on the ontology of biological process, cellular component and molecular function was performed and reveals widely different distribution between *DYRK1A* mRNA and GFP mRNA injection (Supplemental Table S2).

To reveal the nature of the differentially expressed proteins upon *DYRK1A* mRNA and GFP mRNA injection, the GO functional enrichment of differentially quantified proteins was carried out. The biological process was firstly investigated (Fig. 5c), it is found that the up-regulated proteins in response to *DYRK1A* mRNA injection show enrichment of ion, hydrogen, ATP transport. In the down-regulated proteins, the enrichment includes macromolecule biosynthetic process, cellular protein metabolic process and ncRNA metabolic process **(**Fig. 5d).

Molecular function-based enrichment results were shown in Fig. 5c. It is found that the ion/substrate-specific/hydrogen ion transmembrane transporter activity and anion/voltage-gated ion channel activity in up-regulated proteins; and the structural molecule activity and structural constituent of ribosome in down-regulated proteins were enriched in *DYRK1A* mRNA injected embryos **(**Fig. 5d).

In the cellular component category, cytoplasmic part, organelle membrane, mitochondrion, proton-transporting ATPase complex and bounding membrane of organelle were enriched in the up-regulated proteins. In the down-regulated proteins, enrichment includes ribosome, ribonucleoprotein complex and non-membrane-bounded organelle **(**Fig. 5c and d).

**Figure 6:**
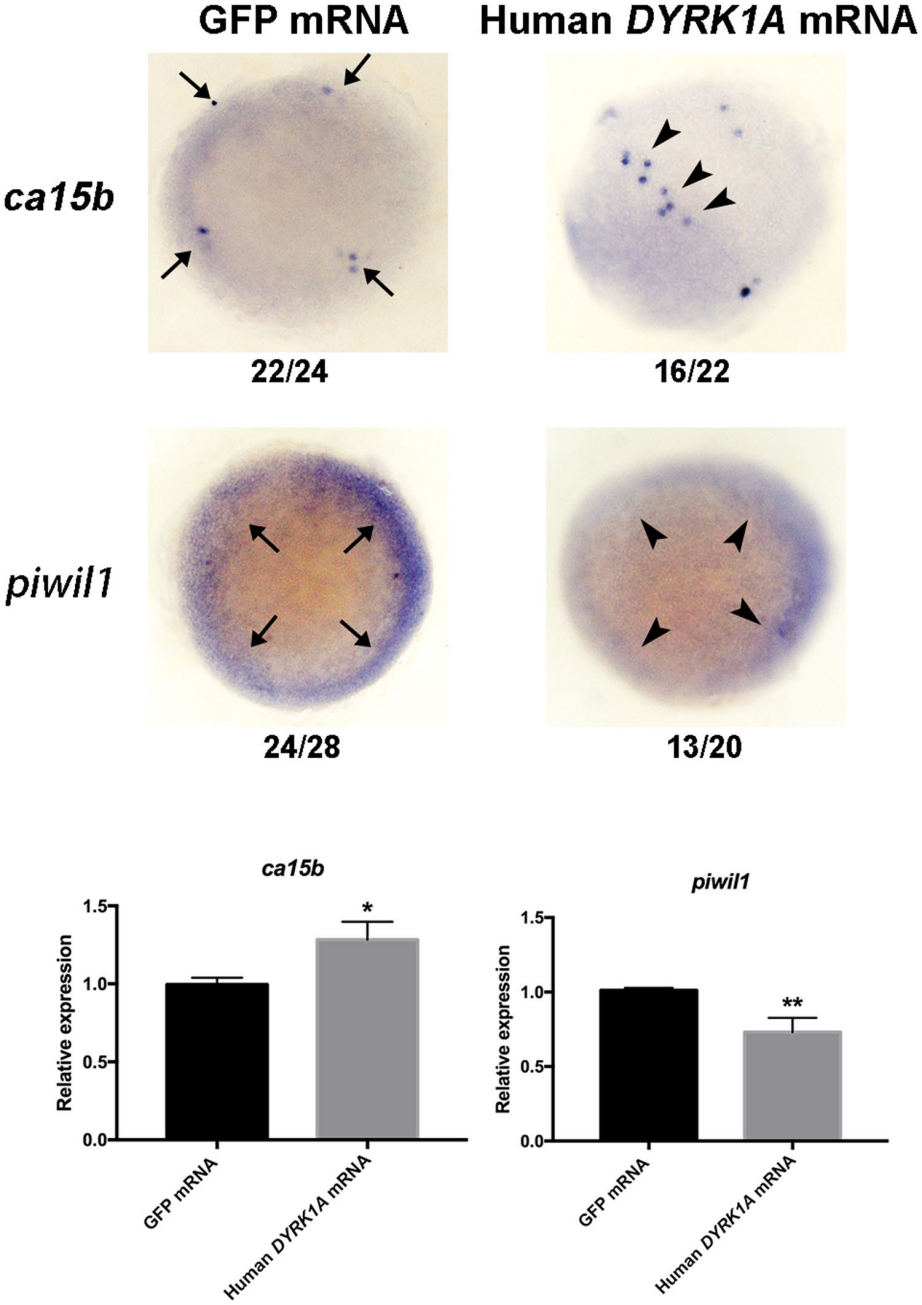
Verification of aberrant expression of PGCs key factors in *DYRK1A* overexpressed embryos. Analysis of localization and strength of *ca15b* and *piwil1* in *DYRK1A* overexpressed embryos by WISH. All of embryos are 50%-epiboly stage with top view. Arrows show the normal location of detected gene, arrowheads demonstrate the aberrant position induced by overexpressed *DYRK1A*. Histogram representing the relative expression of *ca15b and piwil1* in GFP injected and *DYRK1A* overexpressed embryos detected by qPCR.

Of note, we found that expression of *ca15b* (ratio 1.429, P value 0.014260929) and *piwil1* (ratio 0.755, P value 0.014935562) was significantly changed in *DYRK1A* mRNA injected embryos (Supplemental Table S3). Piwil1 is identified as a critical factor essential for PGCs maintenance and migration in zebrafish and medaka^24,27^. Ca15b, an enzyme expressed specifically in the PGCs, plays an important role in establishment of polar pH distribution for guided PGCs migration^25^. Subsequently, we used *in situ* hybridization and qPCR assays to verify the expression of *ca15b* and *piwil1* in *DYRK1A* overexpressed embryos. Results showed that overexpressed *DYRK1A* led to upregulation of *ca15b* and downregulation of *piwil1* indeed, which were consistent with the proteomics data (Fig. 6).

In conclusion, quantitative analysis of global proteome between *DYRK1A* mRNA and GFP mRNA injection indicates that overexpression of *DYRK1A* induces significant impacts in embryos, resulting the remarkably altered expression of many critical proteins for PGCs development, such as Ca15b and Piwil1.

## Discussion

*DYRK1A* gene in human localizes to the Down syndrome critical region q22.2 of chromosome 21, and its overexpression is associated with abnormal phenotype of Down syndrome patients. Down syndrome shows severe defect of gonads and germ cells, however, the distinct function of increased *DYRK1A* dosage in PGCs remains obscured.

Both zebrafish and human *DYRK1A* protein have same PKc_DYRK1 and S_TKc domains, suggesting the evolutional conservation. We injected *DYRK1A* mRNA into embryos and detected the PGCs marker gene *vasa* and *nanos1*. Results demonstrated that overexpressed either zebrafish or human *DYRK1A* induced same abnormity of PGCs, showing reduction and disorder. These results are similar to that observed from trisomy 16 mouse^7^, indicating that overexpression of *DYRK1A* impairs PGCs maintenance and migration, and suggesting the evolutional functional conservation of *DYRK1A* in human and zebrafish.

Subsequently, we performed quantitative proteome analysis to uncover the molecular mechanism of overexpressed *DYRK1A* to PGCs development and early embryogenesis. It is a pity that the identified quantitative proteins are less than expectation, because of the obstacle from the high proportion of yolk proteins in early embryos. Even so, we still identified 1573 proteins and quantified 1324 proteins, and found 265 differentially expressed proteins. GO function classification analysis reveal that wide range of proteins are regulated by overexpressed *DYRK1A*, including transport of ion/hydrogen/ATP, process of macromolecule/protein metabolism/ncRNA metabolism, etc. Most importantly, we identify that *piwil1* and *ca15b* gene expression are significantly changed in *DYRK1A* overexpressed embryos, the two genes are key factors which play critical role in PGCs development process.

Loss of zebrafish *piwil1*(*ziwi*) function results in a progressive loss of germ cells due to apoptosis during larval development^24^. Houwing *et al*. found that wild-type and *piwil1* mutant gonads had similar germ cell numbers at two weeks of age^24^. However, Houwing *et al*. did not show PGCs status of *piwil1* mutant and *piwil1* expression pattern at blastula period of early embryogenesis in zebrafish. Li *et al*. showed that medaka *piwil1* knockdown significantly reduces the number of PGCs *in vivo* and *in vitro* and affects the distribution of PGCs in early developing embryos. Surprisingly, depletion of zygotic medaka *piwil1* severely and specifically affected PGC migration at early embryogenesis^27^. Interestingly, both zebrafish^24^ and medaka^27^ *piwil1* is maternally provided and localizes to granules at the cleavage planes in four cell embryos similar to vasa mRNA. However, *piwil1* RNA was widely distributed to many cells, but not concentrated in PGCs, at the blastula stage in medaka^27^, and we also found the similar expression pattern in zebrafish (Fig. 6), suggesting that *piwil1* may play diverse role in embryogenesis and PGCs development at different stages.

Studies show that the gradient interpretation produces intracellular response, that is, elevation of pH at the front of PGCs drive their migration. Carbonic anhydrase 15b (Ca15b), an enzyme that is expressed specifically in the PGCs, plays an important role in establishment of polar pH distribution for guided PGCs migration. In *ca15b* knockdown embryos, a uniform low pH level is observed within PGCs along with an increased proportion of PGCs that failed to reach their target. At the meantime, lack of chemokine Cxcl12a gradient, reduction of local Rac1 activity and non-polarization of actin occur at the leading edge of the migrating cells in Ca15b knockdown embryos^23,25^. Then, the actin loses to polymerization and leads the loss of myosin activity and normal formation of cellular structures, finally, PGCs migration fail to track correctly^28^.

## Methods

### Ethics Statement

All experiments in this study were in accordance with the “Guide for the Care and Use of Laboratory Animals” (Eighth Edition, 2011. ILARCLS, National Research Council, Washington, D.C.) and were approved by the Animal Care and Use Committee of West China Second University Hospital, Sichuan University (Approval ID: HXDEYY20101105).

### Zebrafish and embryos

Zebrafish WT embryos from AB strain were used. Embryos were obtained by natural mating and cultured in embryo medium^29^. Staging of the embryos was carried out according to Kimmel *et al*.^30^.

### Constructs

Zebrafish *DYRK1A* full length mRNA sequence was from openbiosystems (Catalog Number MDR1734-202804618). The coding region with poly A sequence was amplified by primer pair *zDYRK1A*-cds (Supplemental Table S4) for capped mRNA synthesis; Human *DYRK1A* full length mRNA sequence was amplified by primer pair h*DYRK1A*-cds (Supplemental Table S4) from K562 cells and cloned into pcDNA3.1+ vector (Invitrogen) for capped mRNA synthesis. Fragments of PGCs marker genes *vasa* (GenBank# NM_131057) and *nonos1* (GenBank# NM_131878) (primers are shown in Supplemental Table S4) were cloned into pEASY-T3 (Transgen) for antisense RNA probe synthesis. The ∆PKc domain expression plasmid of *dyrk1a* was constructed using KOD-Plus-Mutagenesis Kit (TOYOBO) using primer pair h*DYRK1A*-∆PKc and z*DYRK1A*-∆PKc (Supplemental Table S4).

### RNA microinjection and reagent treatment

Capped mRNAs were synthesized using mMESSAGE mMACHINE^®^ Kit (Ambion); Synthetic capped mRNAs were injected into single-cell embryos. Injection dose was an estimated amount received by a single embryo, ~30 pg mRNA of *DYRK1A* and GFP was injected into embryos. Epigallocatechin Gallate (EGCG, S2250) was from Selleck. At the beginning of blastula period (2.5 hpf), embryos (30 embryos in a well of 6-well plate with 3 ml culture water) were treated with EGCG for 2.5 h and then subject to whole-mount *in situ* hybridization and quantitative real time RT–PCR.

### Zebrafish embryo *in situ* hybridization and immunohistochemistry

Whole-mount *in situ* hybridization (WISH) was carried out as previously described in Thisse *et al*.^26^ and Sun *et al*.^31^. After lineage by appropriate restriction enzymes, antisense RNAs for *in situ* hybridization were synthesized using DIG RNA Labeling Kit (SP6/T7) (Roche) and purified by MEGAclear (Ambion).

Whole-mount immunohistochemistry (WIHC) in zebrafish embryos was performed as previously described (Jia *et al*.^32^ and Brend *et al*.^33^) with modifications. Embryos were fixed in fresh 2% paraformaldehyde for overnight, permeabilized in 100% methanol at −20 °C for at least 1 hour. The embryos were bathed in PBS solution, then incubated in block solution (PBS plus 0.5% Triton X-100 and 1% BSA) for 1 hr at room temperature. Embryos were then incubated with primary antibody at 4 °C overnight followed incubation with secondary antibodies (HRP conjugate from Thermo Fisher Corporation) at room temperature for 1 hour. DAB kit (ZSGB-BIO) was used to develop color.

### Quantitative real time RT–PCR (qPCR) analysis

Total RNA was prepared with TRIzol (Invitrogen, 15596-018) and cDNA was synthesized from 1 μg of RNA with PrimeScript RT reagent Kit (Takara, DRR037A). qPCR was performed with the SYBR Green detection method with 7500 real-time PCR system (Applied Biosystems). The primers used were shown in Supplemental Table S4.

### Grayscale measurement and statistical analysis

ImageJ software was used to measure signal strength grayscale of whole-mount *in situ* hybridization assay. Statistical analyses were performed with a Student’s t test. Quantitative data show the mean + SD. Statistical significance is defined as *P < 0.05, **P < 0.01, ***P < 0.001.

### Proteomics analysis of embryos

Antibodies for western blot were used: anti-*DYRK1A* (Santa cruz, sc-12568), anti-β-tubulin (Zen Bioscience, 200608). Quantitative proteome analysis was performed by PTM-Biolabs (HangZhou) Co., Ltd., detailed materials and methods are shown in Supplementary Information Materials and Methods.

## Electronic supplementary material

Supplementary Information

Supplementary Dataset 1

Supplementary Dataset 2

Supplementary Dataset 3

Supplementary Dataset 4

## References

[1] Finch RBJA, Finley WH, Finley SC, Tucker CC (1966). Meiosis in trisomic Down’s syndrome. Ala J Med Sci.

[2] Hojager B, Peters H, Byskov AG, Faber M (1978). Follicular development in ovaries of children with Down’s syndrome. Acta Paediatr Scand.

[3] Kjessler B, De la Chapelle A (1971). Meiosis and spermatogenesis in two postpubertal males with Down’s syndrome: 47, XY, G+. Clinical genetics.

[4] Johannisson R (1983). Down’s syndrome in the male. Reproductive pathology and meiotic studies. Hum Genet.

[5] Speed RM (1984). Meiotic configurations in female trisomy 21 foetuses. Hum Genet.

[6] Mittwoch U, Mahadevaiah S, Setterfield LA (1984). Chromosomal anomalies that cause male sterility in the mouse also reduce ovary size. Genet Res.

[7] Leffler A, Ludwig M, Schmitt O, Busch LC (1999). Germ cell migration and early development of the gonads in the trisomy 16 mouse–an animal model for Down’s syndrome. Ann Anat.

[8] Rahmani Z (1989). Critical role of the D21S55 region on chromosome 21 in the pathogenesis of Down syndrome. Proc Natl Acad Sci USA.

[9] Rahmani Z (1990). Down syndrome critical region around D21S55 on proximal 21q22.3. Am J Med Genet Suppl.

[10] Delabar JM (1993). Molecular mapping of twenty-four features of Down syndrome on chromosome 21. Eur J Hum Genet.

[11] Shindoh N (1996). Cloning of a human homolog of the Drosophila minibrain/rat Dyrk gene from “the Down syndrome critical region” of chromosome 21. Biochem Biophys Res Commun.

[12] Song WJ (1996). Isolation of human and murine homologues of the Drosophila minibrain gene: human homologue maps to 21q22.2 in the Down syndrome “critical region”. Genomics.

[13] Lochhead PA, Sibbet G, Morrice N, Cleghon V (2005). Activation-loop autophosphorylation is mediated by a novel transitional intermediate form of DYRKs. Cell.

[14] Kentrup H (1996). Dyrk, a dual specificity protein kinase with unique structural features whose activity is dependent on tyrosine residues between subdomains VII and VIII. The Journal of biological chemistry.

[15] Dierssen M, de Lagran MM (2006). *DYRK1A* (dual-specificity tyrosine-phosphorylated and -regulated kinase 1A): a gene with dosage effect during development and neurogenesis. TheScientificWorldJournal.

[16] Galceran, J., de Graaf, K., Tejedor, F. J. & Becker, W. The MNB/*DYRK1A* protein kinase: genetic and biochemical properties. *Journal of neural transmission. Supplementum*, 139–148 (2003). 10.1007/978-3-7091-6721-2_12 15068246

[17] Hammerle, B., Elizalde, C., Galceran, J., Becker, W. & Tejedor, F. J. The MNB/*DYRK1A* protein kinase: neurobiological functions and Down syndrome implications. *Journal of neural transmission. Supplementum*, 129–137 (2003). 10.1007/978-3-7091-6721-2_11 15068245

[18] Guimera J, Casas C, Estivill X, Pritchard M (1999). Human minibrain homologue (MNBH/DYRK1): characterization, alternative splicing, differential tissue expression, and overexpression in Down syndrome. Genomics.

[19] Arron JR (2006). NFAT dysregulation by increased dosage of DSCR1 and *DYRK1A* on chromosome 21. Nature.

[20] Weidinger G, Wolke U, Koprunner M, Klinger M, Raz E (1999). Identification of tissues and patterning events required for distinct steps in early migration of zebrafish primordial germ cells. Development.

[21] Weidinger G (2002). Regulation of zebrafish primordial germ cell migration by attraction towards an intermediate target. Development.

[22] Sang X, Curran MS, Wood AW (2008). Paracrine insulin-like growth factor signaling influences primordial germ cell migration: *in vivo* evidence from the zebrafish model. Endocrinology.

[23] Paksa A, Raz E (2015). Zebrafish germ cells: motility and guided migration. Curr Opin Cell Biol.

[24] Houwing S (2007). A role for Piwi and piRNAs in germ cell maintenance and transposon silencing in Zebrafish. Cell.

[25] Tarbashevich K, Reichman-Fried M, Grimaldi C, Raz E (2015). Chemokine-Dependent pH Elevation at the Cell Front Sustains Polarity in Directionally Migrating Zebrafish Germ Cells. Curr Biol.

[26] Thisse C, Thisse B (2008). High-resolution *in situ* hybridization to whole-mount zebrafish embryos. Nature protocols.

[27] Li M, Hong N, Gui J, Hong Y (2012). Medaka piwi is essential for primordial germ cell migration. Curr Mol Med.

[28] Jaglarz MK, Howard KR (1995). The active migration of Drosophila primordial germ cells. Development.

[29] Westerfield, M. *The Zebrafish Book*. (University of Oregon Press, 1993).

[30] Kimmel CB, Ballard WW, Kimmel SR, Ullmann B, Schilling TF (1995). Stages of embryonic development of the zebrafish. Dev Dyn.

[31] Sun H (2010). Zili inhibits transforming growth factor-beta signaling by interacting with Smad4. J Biol Chem.

[32] Jia S (2012). Protein phosphatase 4 cooperates with Smads to promote BMP signaling in dorsoventral patterning of zebrafish embryos. Developmental cell.

[33] Brend, T. & Holley, S. A. Zebrafish whole mount high-resolution double fluorescent *in situ* hybridization. *Journal of visualized experiments: JoVE*, 10.3791/1229 (2009). 10.3791/1229 PMC2789764 19322135

