# Supplementary material for: Overexpression of DYRK1A, a Down Syndrome Candidate gene, Impairs Primordial Germ Cells Maintenance and Migration in zebrafish
Source: Sci Rep. 2017 Nov 10;7:15313. doi: 10.1038/s41598-017-15730-w (PMC5681638; doi:10.1038/s41598-017-15730-w)
Supplement: Supplementary file 1 — Supplementary Information [file 41598_2017_15730_MOESM1_ESM.pdf]

Title:

Overexpression of *DYRK1A*, a Down Syndrome Candidate gene, Impairs Primordial Germ Cells Maintenance and Migration in zebrafish

Authors:

Yanyan Liu, Ziyuan Lin, Mingfeng Liu, He Wang, Huaqin Sun

Supplemental Figure S1. Spatiotemporal expression pattern of Dyrk1a protein in zebrafish embryos. Detection of zebrafish Dyrk1a protein using whole-mount immunohistochemistry (WIHC) at indicated stages. Arrows show the stronger expression region of Dyrk1a protein. It is shown that expression pattern of Dyrk1a protein is similar to *dyrk1a* transcripts detected by WISH. Embryo orientations: 2-cell, 30%-epiboly and 50%-epiboly stage, lateral views with the animal pole oriented at the top; 8-somite, Prim-5 and Prim-25 stage, lateral views with anterior oriented toward the left.

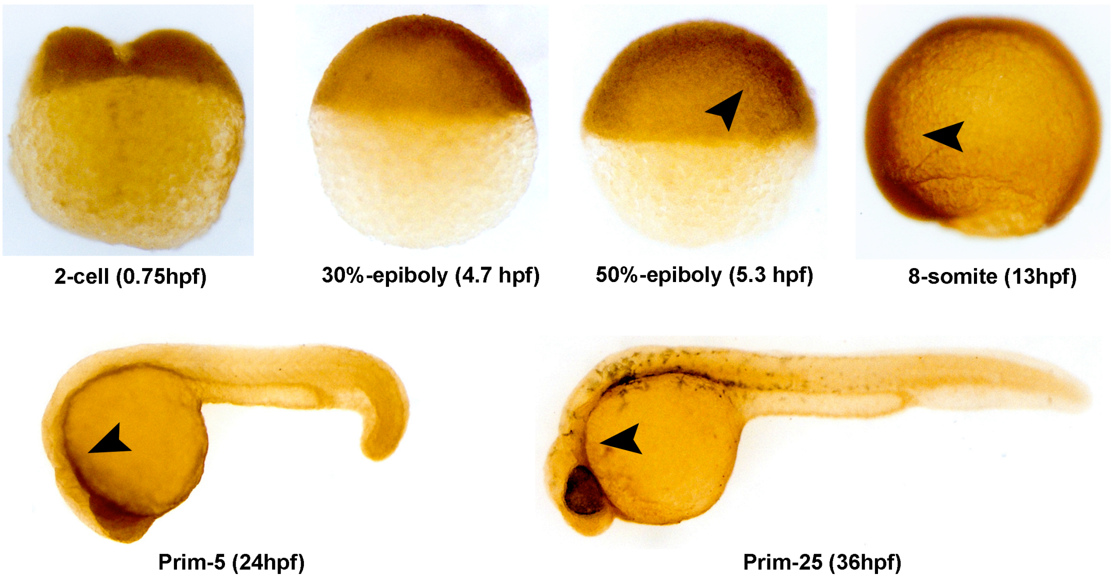

Supplemental Figure S2. PKc conserved domain deletion mutant of DYRK1A fails to induce abnormality of PGCs development. Analysis of localization and strength of *nanos1* and *vasa* by WISH. All of embryos are 50%-epiboly stage with top view. Arrows show the normal location of detected gene. Histogram representing the relative expression of *nanos1* and *vasa* detected by qPCR.

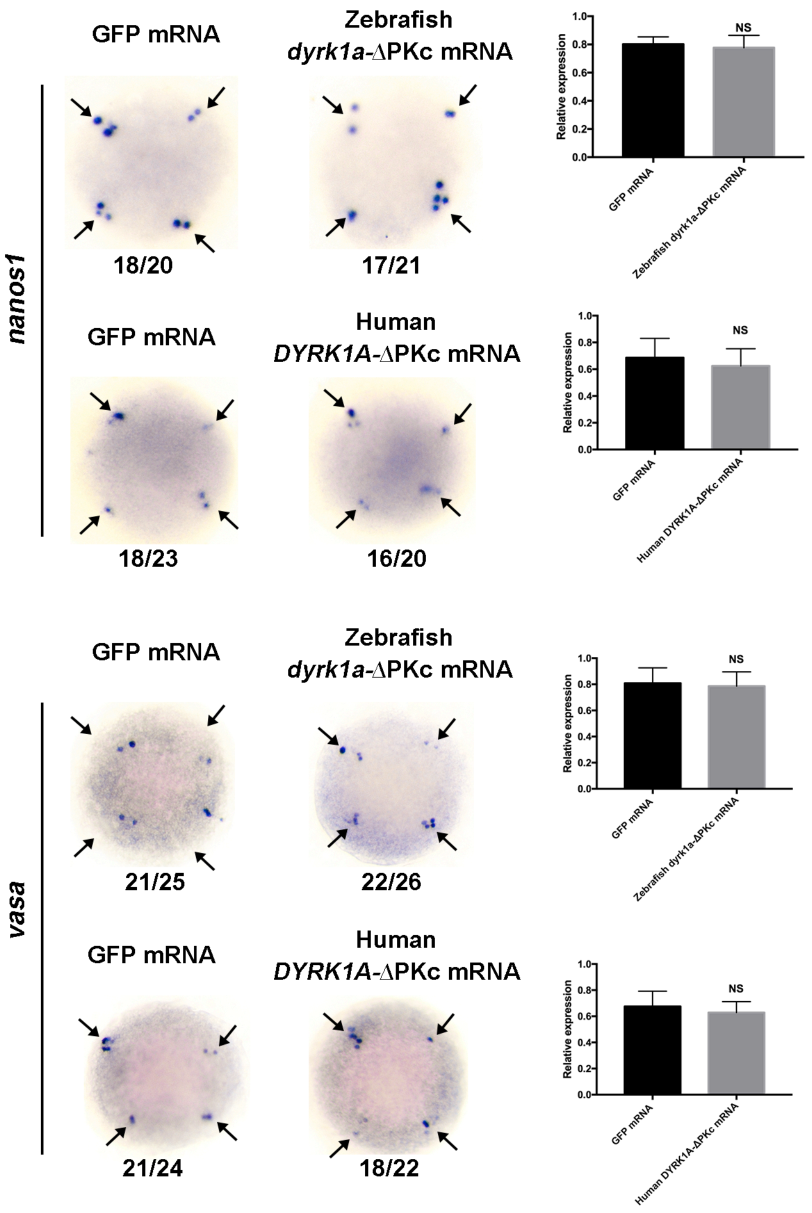

Supplemental Figure S3. Dyrk1a inhibitor EGCG can not lead to aberration of PGCs development. Analysis of localization and strength of *nanos1* and *vasa* by WISH. All of embryos are 50%-epiboly stage with top view. Arrows show the normal location of detected gene. Histogram representing the relative expression of *nanos1* and *vasa* detected by qPCR.

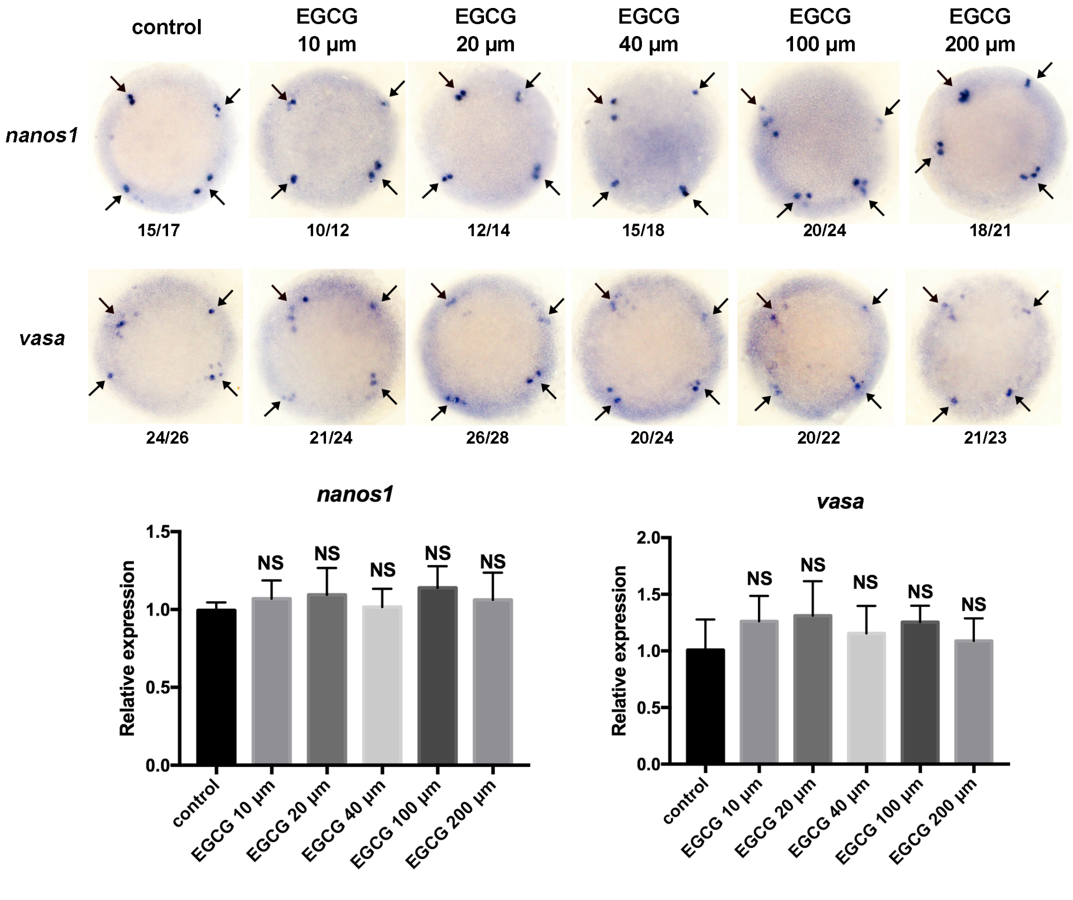

Supplemental Figure S4. Full-length blots for Figure 5b from the main text.

DYRK1A

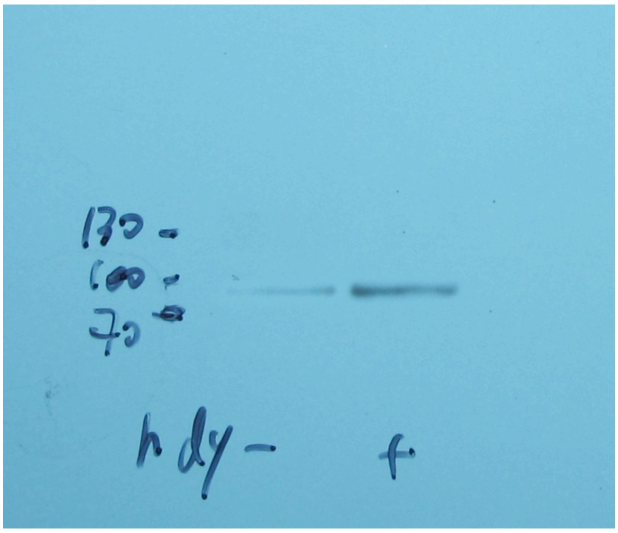

β-tubulin

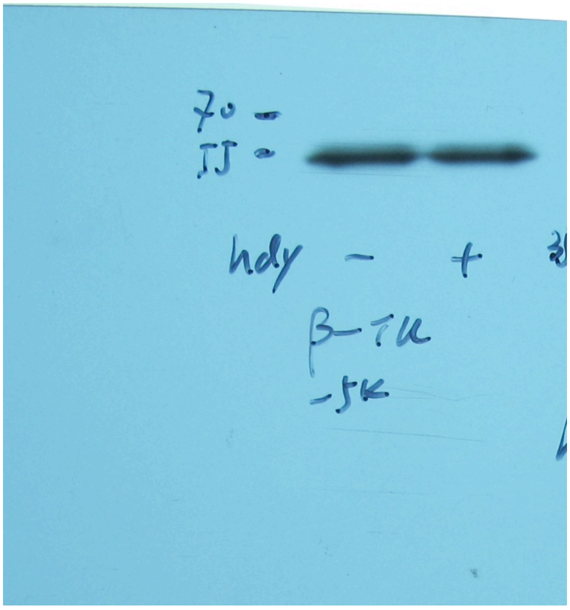

Supplementary Information Materials and Methods

Materials and Methods for Quantitative proteome analysis

1.1. Sample Preparation

1.1.1. Materials and Reagents

Name	Company
TMT Kit	Thermo
Sequencing Grade Modified Trypsin	Promega
EtCH (ethyl alcohol)	Fisher Chemical
ACN (acetonitrile)	Fisher Chemical
TFA (trifluoroacetic acid)	Sigma-Aldrich
FA (formic acid)	Fluka
IAA (iodoacetamide)	Sigma
DTT (dithiothreitol)	Sigma
ProteoMiner^{TM} Protein Enrichment Kit	Bio-Rad
2-D Quant kit	GE Healthcare

1.1.2. Protein Extraction

Sample was sonicated three times on ice using a high intensity ultrasonic processor (Scientz) in lysis buffer (8 M urea, 2 mM EDTA, 10 mM DTT and 1% Protease Inhibitor Cocktail). The remaining debris was removed by centrifugation at 20,000g at 4 °C for 10 min. Finally, the protein was precipitated with cold 15% TCA for 2 h at -20 °C. After centrifugation at 4 °C for 10 min, the supernatant was discarded. The remaining precipitate was washed with cold acetone for three times. The protein was redissolved in buffer (8 M urea, 100 mM TEAB, pH 8.0) and the protein concentration was determined with 2-D Quant kit according to the manufacturer's instructions. Additionally, in each sample (2 mg), high abundant proteins were removed using ProteoMiner^{TM} Protein Enrichment Kit, and 60 µl solution were obtained in each sample. Protein concentration was determined again with 2-D Quant kit according to the manufacturer's instructions.

1.1.3. Trypsin Digestion

For digestion, the protein solution was reduced with 10 mM DTT for 1 h at 37 °C and alkylated with 20 mM IAA for 45 min at room temperature in darkness. For trypsin digestion, the protein sample was diluted by adding 100 mM TEAB to urea concentration less than 2M. Finally, trypsin was added at 1:50 trypsin-to-protein mass ratio for the first digestion overnight and 1:100 trypsin-to-protein mass ratio for a second 4 h-digestion. Approximately 100 μg protein for each sample was digested with trypsin for the following experiments.

1.1.4. TMT Labeling

After trypsin digestion, peptide was desalted by Strata X C18 SPE column (Phenomenex) and vacuum-dried. Peptide was reconstituted in 0.5 M TEAB and processed according to the manufacturer's protocol for 6-plex TMT kit. Briefly, one unit of TMT reagent (defined as the amount of reagent required to label 100 μg of protein) were thawed and reconstituted in 24 μl ACN. The peptide mixtures were then incubated for 2 h at room temperature and pooled, desalted and dried by vacuum centrifugation.

Table. Labeling information

Sample Groups	Labeling information
GFP	128
Dyrk1a	130

1.1.5. HPLC Fractionation

The sample was then fractionated into fractions by high pH reverse-phase HPLC using Agilent 300Extend C18 column (5 μm particles, 4.6 mm ID, 250 mm length). Briefly, peptides were first separated with a gradient of 2% to 60% acetonitrile in 10 mM ammonium bicarbonate pH 10 over 80 min into 80 fractions. Then, the peptides were combined into 18

fractions and dried by vacuum centrifuging.

1.2. Quantitative Proteomic Analysis by LC-MS/MS

1.2.1. Materials and Reagents

Name	Company
H_2O	Thermo
ACN (acetonitrile)	Fisher Chemical
FA (formic acid)	Fluka

Mass Spectrometer:

Thermo Scientific™ Orbitrap Fusion™

1.2.2. LC-MS/MS Analysis

Peptides were dissolved in 0.1% FA, directly loaded onto a reversed-phase pre-column (Acclaim PepMap 100, Thermo Scientific). Peptide separation was performed using a reversed-phase analytical column (Acclaim PepMap RSLC, Thermo Scientific). The gradient was comprised of an increase from 6% to 25% solvent B (0.1% FA in 98% ACN) over 42 min, 25% to 40% in 12 min and climbing to 80% in 3 min then holding at 80% for the last 3 min, all at a constant flow rate of 350 nl/min on an EASY-nLC 1000 UPLC system, The resulting peptides were analyzed by Orbitrap Fusion™ hybrid quadrupole-Orbitrap mass spectrometer (ThermoFisher Scientific).

The peptides were subjected to NSI source followed by tandem mass spectrometry (MS/MS) in Orbitrap Fusion™ (Thermo) coupled online to the UPLC. Intact peptides were detected in the Orbitrap at a resolution of 60,000. Peptides were selected for MS/MS using NCE setting as 38; ion fragments were detected in the Orbitrap at a resolution of 15,000. A data-dependent procedure that alternated between one MS scan followed by MS/MS scans at top speed was applied for the top abundant precursor ions above a threshold ion count of 1E4 in the MS survey scan with 30.0 s dynamic exclusion. The electrospray voltage applied was 2.0 kV. Automatic gain control (AGC) was used to prevent overfilling of the Orbitrap; 1E5 ions were

accumulated for generation of MS/MS spectra. For MS scans, the m/z scan range was 400 to 1600. Fixed first mass was set at 100 m/z.

1.2.3. Database Search

The resulting MS/MS data were processed using Maxquant search engine (v.1.5.1.8). Tandem mass spectra were searched against *Uniprot Danio Rerio* database. Trypsin/P was specified as cleavage enzyme allowing up to 2 missing cleavages. Mass error was set to 10 ppm for precursor ions and 0.02 Da for fragment ions. Carbamidomethyl on Cys, were specified as fixed modification and oxidation on Met was specified as variable modifications. For protein quantification method, TMT-6-plex was selected in Mascot. FDR was adjusted to < 1% and peptide ion score was set ≥ 20.

1.2.4. QC Validation of MS Data

The MS data validation was shown in following **Figure**. The length of most peptides distributed between 8 and 16, which agree with the property of tryptic peptides, that means sample preparation reach the standard (**A**). The log_2-transformed dyrk1a/GFP ratio distribution approximately fits normal distribution, which agrees with the nature of this type of data (**B**).

A.

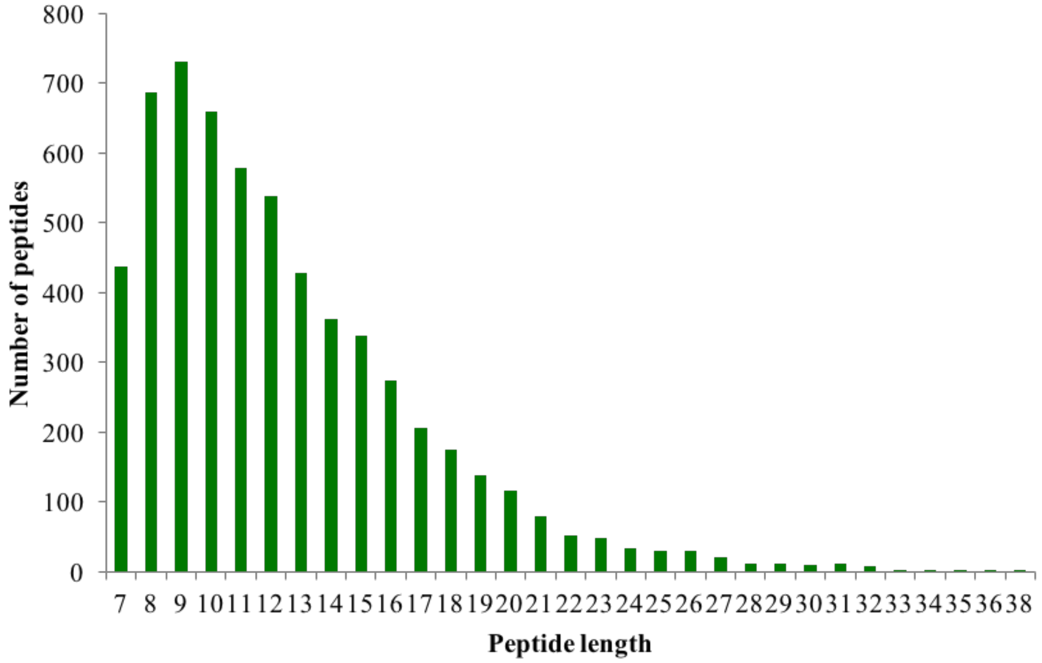

B.

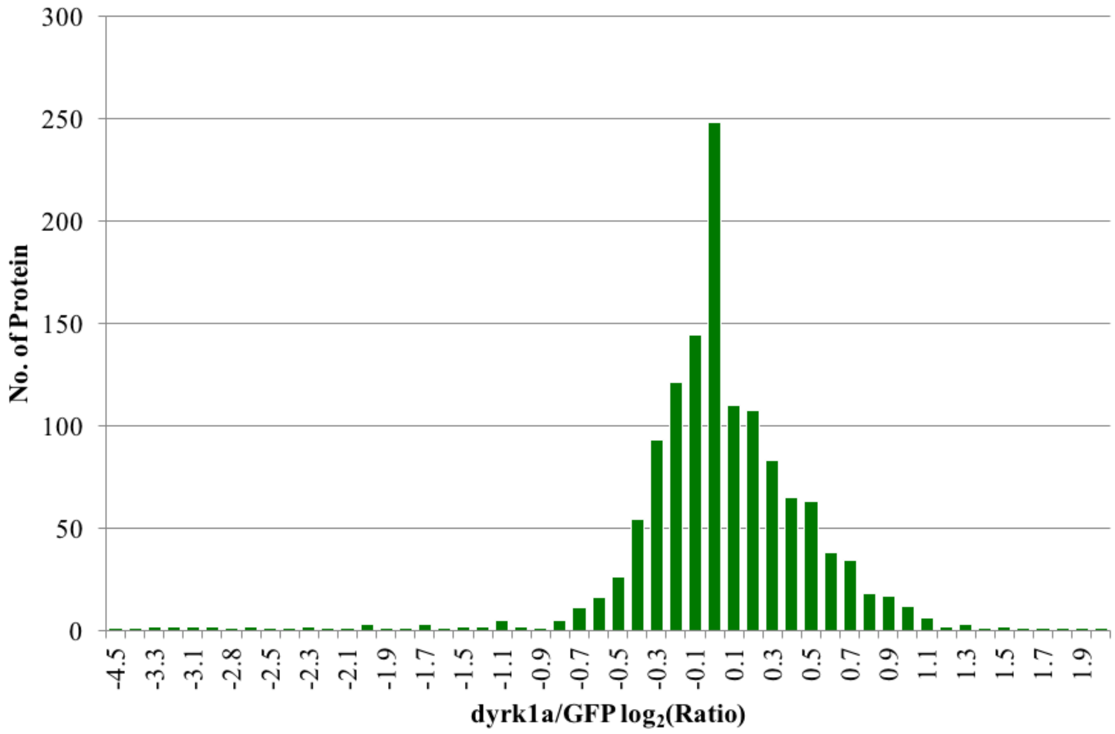

Figure. QC validation of MS data: Peptide length distribution (A) and $\log_2$-transformed ratio distribution (B).

1.3. Bioinformatics Methods

1.3.1. Annotation Methods

GO Annotation

The Gene Ontology, or GO, is a major bioinformatics initiative to unify the representation of gene and gene product attributes across all species. More specifically, the project aims to:

1. Maintain and develop its controlled vocabulary of gene and gene product attributes;
2. Annotate genes and gene products, and assimilate and disseminate annotation data;
3. Provide tools for easy access to all aspects of the data provided by the project.

The ontology covers three domains:

1. Cellular component: A cellular component is just that, a component of a cell, but with the proviso that it is part of some larger object; this may be an anatomical structure (e.g. rough endoplasmic reticulum or nucleus) or a gene product group (e.g. ribosome, proteasome or a protein dimer).
2. Molecular function: Molecular function describes activities, such as catalytic or binding activities, that occur at the molecular level. GO molecular function terms represent activities rather than the entities (molecules or complexes) that perform the actions, and do not specify where or when, or in what context, the action takes place.
3. Biological process: A biological process is series of events accomplished by one or more ordered assemblies of molecular functions. It can be difficult to distinguish between a biological process and a molecular function, but the general rule is that a process must have more than one distinct steps.

Gene Ontology (GO) annotation proteome was derived from the UniProt-GOA database (www. http://www.ebi.ac.uk/GOA/). Firstly, Converting identified protein ID to UniProt ID and then mapping to GO IDs by protein ID. If some identified proteins were not annotated by UniProt-GOA database, the InterProScan soft would be used to annotated protein's GO functional based on protein sequence alignment method. Then proteins were classified by Gene Ontology annotation based on three categories: biological process, cellular component

and molecular function.

Domain Annotation

A protein domain is a conserved part of a given protein sequence and structure that can evolve, function and exist independently of the rest of the protein chain. Each domain forms a compact three-dimensional structure and often can be independently stable and folded. Many proteins consist of several structural domains. One domain may appear in a variety of different proteins. Molecular evolution uses domains as building blocks and these may be recombined in different arrangements to create proteins with different functions. Domains vary in length from between about 25 amino acids up to 500 amino acids in length. The shortest domains such as zinc fingers are stabilized by metal ions or disulfide bridges. Domains often form functional units, such as the calcium-binding EF hand domain of calmodulin. Because they are independently stable, domains can be "swapped" by genetic engineering between one protein and another to make chimeric proteins.

Identified proteins domain functional description were annotated by InterProScan (a sequence analysis application) based on protein sequence alignment method, and the InterPro domain database was used. InterPro (http://www.ebi.ac.uk/interpro/) is a database that integrates diverse information about protein families, domains and functional sites, and makes it freely available to the public via Web-based interfaces and services. Central to the database are diagnostic models, known as signatures, against which protein sequences can be searched to determine their potential function. InterPro has utility in the large-scale analysis of whole genomes and meta-genomes, as well as in characterizing individual protein sequences.

KEGG Pathway Annotation

KEGG connects known information on molecular interaction networks, such as pathways and complexes (the "Pathway" database), information about genes and proteins generated by genome projects (including the gene database) and information about biochemical compounds and reactions (including compound and reaction databases). These databases are different networks, known as the "protein network", and the "chemical universe" respectively.

There are efforts in progress to add to the knowledge of KEGG, including information regarding ortholog clusters in the KEGG Orthology database. KEGG Pathways mainly including: Metabolism, Genetic Information Processing, Environmental Information Processing, Cellular Processes, Rat Diseases, Drug development. Kyoto Encyclopedia of Genes and Genomes (KEGG) database was used to annotate protein pathway. Firstly, using KEGG online service tools KAAS to annotated protein's KEGG database description. Then mapping the annotation result on the KEGG pathway database using KEGG online service tools KEGG mapper.

Subcellular Localization

The cells of eukaryotic organisms are elaborately subdivided into functionally distinct membrane bound compartments. Some major constituents of eukaryotic cells are: extracellular space, cytoplasm, nucleus, mitochondria, Golgi apparatus, endoplasmic reticulum (ER), peroxisome, vacuoles, cytoskeleton, nucleoplasm, nucleolus, nuclear matrix and ribosomes.

Bacteria also have subcellular localizations that can be separated when the cell is fractionated. The most common localizations referred to include the cytoplasm, the cytoplasmic membrane (also referred to as the inner membrane in Gram-negative bacteria), the cell wall (which is usually thicker in Gram-positive bacteria) and the extracellular environment. Most Gram-negative bacteria also contain an outer membrane and periplasmic space. Unlike eukaryotes, most bacteria contain no membrane-bound organelles, however there are some exceptions.

There, we used Wolfpsort, a subcellular localization predication soft to predict subcellular localization.

1.3.2. Functional Enrichment

Enrichment of Gene Ontology analysis

Proteins were classified by GO annotation into three categories: biological process, cellular compartment and molecular function. For each category, a two-tailed Fisher's exact test was

employed to test the enrichment of the differentially expressed protein against all identified proteins. Correction for multiple hypothesis testing was carried out using standard false discovery rate control methods. The GO with a corrected p-value < 0.05 is considered significant.

Enrichment of pathway analysis

Encyclopedia of Genes and Genomes (KEGG) database was used to identify enriched pathways by a two-tailed Fisher's exact test to test the enrichment of the differentially expressed protein against all identified proteins. Correction for multiple hypothesis testing was carried out using standard false discovery rate control methods. The pathway with a corrected p-value < 0.05 was considered significant. These pathways were classified into hierarchical categories according to the KEGG website.

Enrichment of protein domain analysis

For each category proteins, InterPro (a resource that provides functional analysis of protein sequences by classifying them into families and predicting the presence of domains and important sites) database was researched and a two-tailed Fisher's exact test was employed to test the enrichment of the differentially expressed protein against all identified proteins. Correction for multiple hypothesis testing was carried out using standard false discovery rate control methods and domains with a corrected p-value < 0.05 were considered significant.

1.3.3. Enrichment-based Clustering

Functional Enrichment-based Clustering for Quantitative Category

The quantifiable proteins in this study were divided into four quantitative categories according to their dyrk1a/GFP ratios: Q1 (0< Ratio ≤1/1.5), Q2 (1/1.5 < Ratio≤ 1/1.2), Q3 (1.2 < Ratio≤1.5) and Q4 (Ratio >1.5). Then, the quantifiable proteins from the four categories were plotted for GO enrichment-based cluster analysis

Clustering Method: All the substrates categories obtained after enrichment were collated along with their P values, and then filtered for those categories which were at least enriched

in one of the clusters with P value < 0.05. This filtered P value matrix was transformed by the function $x = -\log10$ (P value). Finally these x values were z-transformed for each category. These z scores were then clustered by one-way hierarchical clustering (Euclidean distance, average linkage clustering) in Genesis. Cluster membership was visualized by a heat map using the “heatmap.2” function from the “gplots” R-package.

Supplementary Information

Supplemental Table S1. Protein annotation from proteomics analysis.

Supplemental Table S2. The GO distribution of up/down-regulated proteins (*DYRK1A* mRNA vs GFP mRNA).

Supplemental Table S3. All of the differentially quantified expressed proteins (*p*<0.05) (*DYRK1A* mRNA vs GFP mRNA).

Supplemental Table S4. Oligonucleotide primers and probes used in the study.
